# Host Plant Dependence of the Symbiotic Microbiome of the Gall-Inducing Wasp *Trichagalma acutissimae*

**DOI:** 10.3390/insects16070652

**Published:** 2025-06-23

**Authors:** Yingnan Wang, Yuanchen Zhang, Ran Li, Yujian Li, Muha Cha, Xianfeng Yi

**Affiliations:** 1 School of Life Sciences, Qufu Normal University, Qufu 273165, China; rosebud269@163.com (Y.W.); li471329014@163.com (R.L.); yujian528@163.com (Y.L.); 2 College of Biological and Food Engineering, Anyang Institute of Technology, Anyang 455000, China; zhangyc2011@163.com

**Keywords:** gall wasp, host plant species, oak, symbiotic microbiota, tannin

## Abstract

Symbiotic bacteria play a pivotal role in the biology and ecology of herbivorous insects, affecting host growth and adaptation. However, the effects of host identity on the symbiotic microbiota of gall-inducing insects remain less explored. In this study, we utilized high-throughput sequencing to investigate the effects of different oak hosts on the structure and diversity of the symbiotic microbial community in the asexual larvae of the gall-inducing wasp *Trichagalma acutissimae*. Host plant species significantly altered the alpha and beta diversity of symbiotic microbiota of *T. acutissimae*. At the phylum level, Proteobacteria was the predominant microflora in both groups, with significantly higher abundance in larvae parasitizing *Quercus acutissima* than in those parasitizing *Q. variabilis*. *Pseudomonas*, which has been identified as responsible for tannin decomposition, was the most dominant genus in *T. acutissimae* larvae infesting both hosts. LEfSe analysis revealed substantial differences in the symbiotic microbial communities between the two hosts while also highlighting some commonalities. Functional prediction analysis indicated no significant difference in the functional roles of symbiotic bacteria between larvae infesting the two hosts. These findings suggest that the symbiotic microbiome of *T. acutissimae* larvae is influenced by host plant species, yet different microbial compositions may perform similar functions, implying the potential role of symbiotic microbiota in the adaptation to high-tannin oak leaves. This research enhances our understanding of the symbiotic relationship between forest pests and their associated microbes.

## Simple Summary

Symbiotic bacteria are vital for herbivorous insects, yet how host plants shape gall wasp microbiota remains unclear. Using high-throughput sequencing, we found that oak species (*Q. acutissima* vs. *Q. variabilis*) altered the microbiome diversity of gall-inducing *Trichagalma acutissimae* larvae. Proteobacteria dominated, with higher abundance in larvae infested *Q. acutissima*, while tannin-degrading *Pseudomonas* was prevalent in both. Despite compositional differences, functional predictions showed similar bacterial roles, suggesting that microbiome flexibility aids adaptation to tannin-rich hosts. This study clarifies host–microbe dynamics in gall wasp *T. acutissimae*.

## 1. Introduction

Symbiotic microbes in insects play an essential role in the host’s biological system, forming a complex symbiotic network with host insects [1,2]. This symbiotic relationship not only affects nutritional metabolism, immune regulation, growth and development, reproduction, and other physiological processes of insects but also enhances their adaptability and evolutionary potential to environmental changes [3,4,5]. For example, gut microbes help insects break down complex organic matter such as cellulose and lignin [6], improving nutrient absorption efficiency. Additionally, symbiotic microbiota produces beneficial metabolites like vitamins and amino acids, meeting the growth needs of insects. When insects feed on different hosts, the symbiotic microbiota influences nutrient absorption and hormone release, thereby affecting growth and development [7,8,9]. Although progress has been made in studying insect gut microbes, many challenges remain. The complexity of insect gut microbial communities complicates research efforts, and the microbial composition varies widely among different insect individuals and environmental conditions [10,11]. The interaction mechanisms between insect gut microbes and their hosts are intricate, involving various signaling molecules and regulatory networks [12,13]. For instance, there are complex interactions between gut microbes and the host’s immune, nervous, and endocrine systems [14]. Analyzing these interaction mechanisms requires interdisciplinary integration and comprehensive research.

The host plant significantly influences the symbiotic microbiota community of insects. Different host plants provide varying nutrients, chemicals, and environmental conditions, affecting the composition and function of insect symbiotic microbiota [15,16]. Secondary metabolites in plants, such as tannins and alkaloids, exert selective pressure on insect gut microbes, screening for microorganisms that can tolerate or degrade these substances [17,18]. Moreover, plant nutrients like carbohydrates, proteins, and fats influence the metabolic activities and growth of insect gut microbes [19]. Under natural conditions, insects and their associated microbial communities adapt under stress, shaping new adaptations through dietary habits and host factors, thereby reducing survival stress [1,20,21]. Studies have shown that insect microbial communities affect the adaptation of insect-feeding species, leading to changes in insect behavior and population ecology [22,23,24]. Significant differences in gut microbial communities have been observed in insects feeding on different host plants, impacting nutrient absorption, growth, adaptability, and selectivity [25,26]. Therefore, understanding the influence of host plants on insect microbes is crucial for comprehending the interaction between insects and plants and the ecological adaptability of insects.

Insect-associated microbiota plays a pivotal role in host physiology, nutrient acquisition, and defense against pathogens, often influencing insect ecology and evolution [27]. Host plants can substantially shape microbial composition by modifying the nutritional environment or introducing plant-specific metabolites [23], although the extent of these effects varies among insect taxa [28]. Gall wasps (Cynipidae) offer an exemplary system for investigating such interactions, as their larvae develop within plant-induced galls, forming a distinct microhabitat that may selectively filter or enrich specific microbial symbionts [29]. *Trichagalma acutissimae* is a gall wasp that primarily infests *Q. variabilis* and *Q. acutissima* (Figure S1). Evidence shows that the asexual larvae of *T. acutissimae* induce galls on leaves, while sexual larvae form galls on male inflorescence the two oak species [30]. While some gall wasp–microbe symbioses have been characterized, the microbiota associated with *T. acutissimae* remain largely unexplored, despite their potential to elucidate how gall-specific environments structure microbial communities. To clarify the correlation between host species and gall inducers is essential for understanding the role of plant-mediated effects in insect–microbe interactions, particularly in specialized gall systems where microbial symbionts may contribute to gall formation, nutrient provisioning, or host defense [31]. Given the ecological significance of gall wasps as both plant parasites and ecosystem engineers, unraveling these relationships could yield broader insights into the mechanisms governing insect–plant–microbe tripartite associations.

Recent advances in molecular biology, particularly high-throughput sequencing technology, have significantly advanced the study of insect symbiotic bacteria. 16S rRNA sequencing technology is widely used to analyze microbial community composition and is the preferred method for studying symbiotic microbiomes. Researchers can now gain deeper insights into the composition, structure, and function of insect symbiotic microbiomes, revealing their important roles in insect physiology and ecology. With technological advancements, high-throughput sequencing, especially Illumina technology, has become the most mature metagenomics tool, capable of analyzing microbial community structures and relative abundances in complex environments [32]. This technology has been extensively applied to study microbial differences in insects [33,34]. To investigate changes in the symbiotic bacteria of *T. acutissimae* larvae when fed on different hosts, we sequenced the 16S rRNA gene using Illumina technology to elucidate the microbiome structure of larvae parasitizing *Q. variabilis* and *Q. acutissima*. We aimed to study the effects of host plants on the symbiotic microbiome of *T. acutissimae* larvae, which is closely related to the biology and ecology of the host insect and lays a foundation for developing green and efficient pest control measures.

## 2. Materials and Methods

### 2.1. Sampling and Sample Processing

Cynipid galls induced by *T. acutissimae* were directly collected from oak trees (*Quercus variabilis* and *Q. acutissima*) located in Shimenshan Town, Qufu City, China. Five trees of each oak species were randomly selected from the same geographic location. From each tree, we collected >50 mature galls with similar size and shape. Galls were transported to the lab and sterilized by using 75% alcohol for 5 min. Then, the larvae were carefully extracted using sterilized forceps from the composite galls, and subsequently allocated into 10 sterile tubes per species, with each tube containing 20 larvae to form a single biological replicate. Because the larvae were contained in the inner capsules suspended by connective tissue in the intact roly-poly galls, our thorough sterilization procedures are expected to minimize some background contamination. Due to the larvae’s small size or the constraints to dissect them, whole larvae were utilized for testing symbiotic microbiota bacteria, instead of gut microbiota. Ten samples of *T. acutissimae* larvae infesting *Q. variabilis* were collected in June 2023, while another ten samples of larvae infesting *Q. acutissima* were collected in July 2023, due to the natural phenological difference in *T. acutissimae* developing on leaves of *Q. acutissima* and *Q. variabilis*. The one-month difference in collection time may incur seasonal shifts in microbiota but may also avoid the influence of developing stages of larvae on the symbiotic microbiota. All samples were immediately frozen in liquid nitrogen after cleaning and stored at −80 °C for subsequent analysis.

### 2.2. Sequencing and Bioinformatics Analysis

DNA was extracted from the whole body of *T. acutissimae* larvae using the QIAamp Fast DNA Stool Mini Kit (QIAGEN, Hilden, Germany) following the manufacturer’s instructions. The quality and integrity of the genomic DNA were verified by agarose gel electrophoresis. Barcode-containing primer adapters were designed and synthesized. PCR amplification was performed using the TransGen AP221-02: TransStart Fastpfu DNA Polymerase system (AP221–02, TransGen, Beijing, China), with three technical replicates per sample. The PCR products from each sample were pooled and subjected to 2% agarose gel electrophoresis for verification. Gel-purified PCR products were recovered using the AxyPrep DNA Gel Recovery Kit (Corning, Glendale, CA, USA) and eluted in a Tris-HCl buffer. After confirming the concentration via 2% agarose gel electrophoresis, the PCR products were quantified using the QuantiFluor™-ST blue fluorescence system (Promega, Madison, WI, USA) and normalized to ensure equal representation. Purified PCR products were used to construct a single-stranded DNA library, which was sequenced to obtain template DNA fragment sequences. Paired-end reads generated from sequencing were assembled based on overlap relationships, followed by quality control and filtering. Sample demultiplexing was performed, and operational taxonomic unit (OTU) clustering and taxonomic classification were conducted. Subsequently, various diversity analyses, including Alpha and Beta diversity analyses, inter-group species difference analysis, association and model prediction analysis, and functional prediction analysis, were performed. This entire process was carried out by Shanghai Majorbio Biomedical Technology Co., Ltd.

### 2.3. Data Analysis

To detect the differences in bacterial alpha diversity, four indices (Ace, Shannon, Chao, and Simpson) were calculated for each sample according to the UPARSE pipeline [35]. The statistical significance of alpha diversity indices was tested using the Kruskal–Wallis test. To assess the variation in microbial composition across the samples, we performed beta diversity analyses on the platform of Majorbio Cloud Platform (cloud.majorbio.com), including the principal coordinate analysis (PCoA) based on the unweighted and weighted UniFrac distances, as well as Non-metric multidimensional scaling (NMDS).

In addition to assessing alpha (α) and beta (β) diversity, LEfSe Analysis (Linear Discriminant Analysis Effect Size) was conducted to identify distinctive microbial communities within the samples [36]. PICRUSt2 functional prediction was employed to predict the functional information of microbial communities in larval samples, thereby facilitating a deeper understanding of certain potential microbial functional characteristics during environmental changes through the analysis of functional composition and abundance.

## 3. Results

### 3.1. Sequencing Data Quality Assessment

The bacterial communities associated with *T. acutissimae* larvae parasitizing *Q. acutissima* and *Q. variabilis* were sequenced using the Illumina HiSeq 2500 platform, yielding a total of 5,671,177 raw reads. After the paired-end read assembly and quality control filtering, 5,080,864 high-quality sequences (90% of the initial reads) were retained for downstream analysis, with an assembly success rate of 89.59%. The average sequence length across all samples was 417 bp, ranging from 400 to 440 bp. Both the Pan, Core, and rarefaction-coverage curves gradually plateaued with increasing sample size, indicating robust sample quality, adequate sequencing depth, and high reliability of subsequent analyses (Figure S2).

### 3.2. Composition and Structure of Symbiotic Bacteria Communities of T. acutissimae

**Figure 1:**
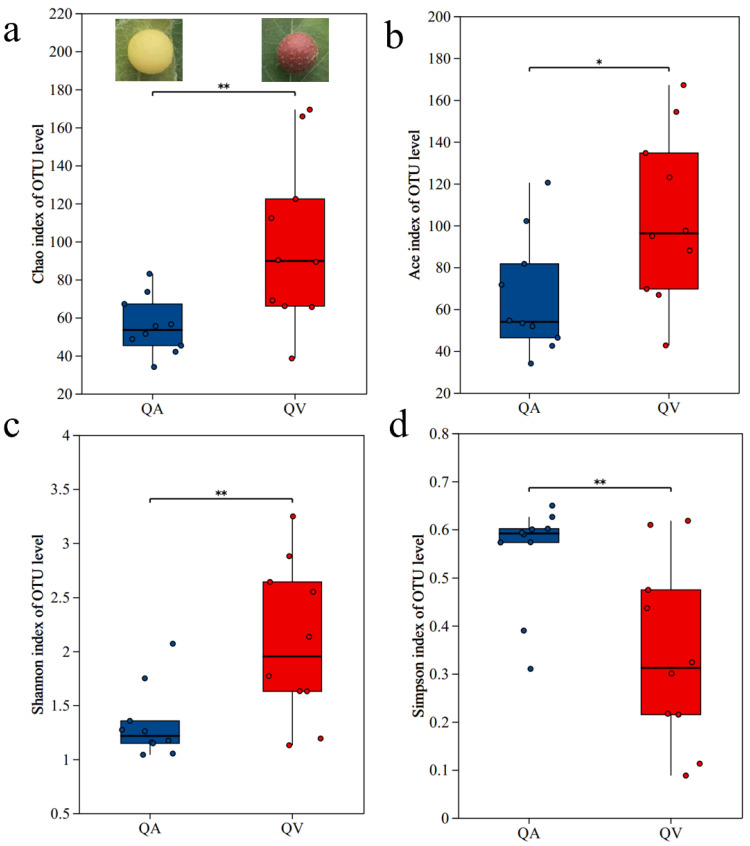
Alpha-diversity indices of the symbiotic microbiota of gall wasp *Trichagalma acutissimae* parasitizing two oak species: (**a**): Ace index; (**b**): Chao index; (**c**): Shannon index; (**d**): Simpson index. QA and QV stand for *Quercus acutissima* and *Q. variabilis*, respectively. * and ** stand for statistically significant at *p* < 0.05 and *p* < 0.01 level, respectively.

Symbiotic bacteria of *T. acutissimae* larvae parasitizing *Q. variabilis* exhibited higher Ace, Chao, and Shannon indices but a lower Simpson index compared to those on *Q. acutissima* (*p* = 0.024; *p* = 0.008; *p* = 0.008; *p* = 0.007) (Figure 1a–d).

**Figure 2:**
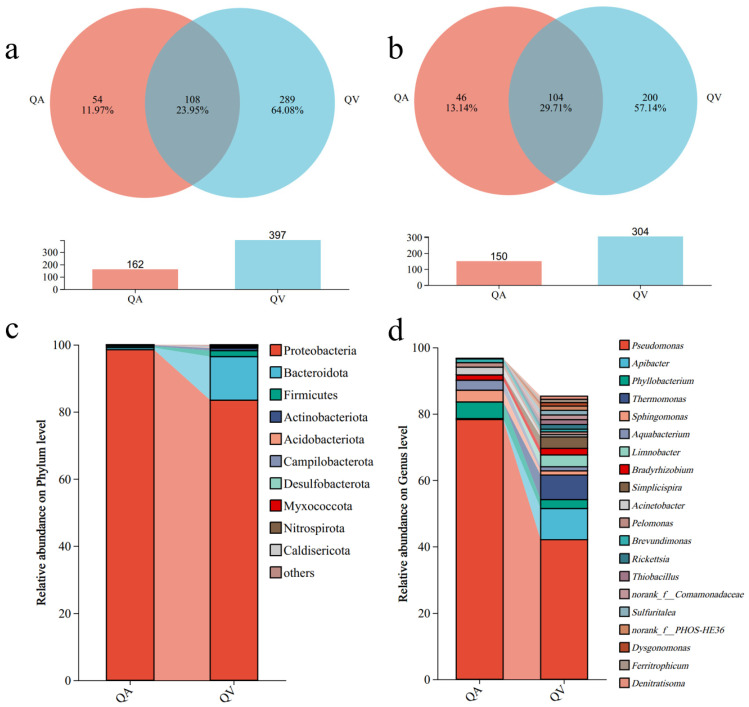
Community composition of the symbiotic microbiota of gall wasp *Trichagalma acutissimae* parasitizing two oak species: (**a**): Venn diagram at the OUT level; (**b**): Venn diagram at the species level; (**c**): Relative abundance at the phylum level; (**d**): Relative abundance at the genus level. QA and QV stand for *Quercus acutissima* and *Q. variabilis*, respectively.

The symbiotic bacteria of *T. acutissimae* larvae exhibited a total of 451 operational taxonomic units (OTUs). Among these, 54 and 289 OTUs were unique to larvae parasitizing *Q. acutissima* and *Q. variabilis*, respectively. We detected 108 OTUs shared between the larvae parasitizing the two hosts (Figure 2a). At the species level, the two groups collectively harbored 350 bacterial species, with 46 and 200 species exclusive to *Q. acutissima* and *Q. variabilis*, respectively. A core microbiome of 104 species was shared between the groups, accounting for 29.71% of the species pool (Figure 2b).

At the phylum level (Figure 2c), the symbiotic microbiota of larvae parasitizing *Q. variabilis* was primarily composed of Proteobacteria (80.70%), *Bacteroidota* (11.95%), Firmicutes (3.07%), and *Actinobacteriota* (2.91%). In contrast, the larvae parasitizing *Q. acutissima* harbored a less diverse community, mainly comprising Proteobacteria (94.15%), Firmicutes (2.59%), and Actinobacteriota (2.62%). At the genus level (Figure 2d), the symbiotic microbiota of larvae parasitizing *Q. variabilis* was predominantly represented by 15 genera, with *Pseudomonas* (45.21%) being the most abundant, followed by *Apibacter* (8.21%) and *Rickettsia* (4.66%). Other notable genera included *Phyllobacterium* (2.88%), *Bartonella* (2.21%), and *Sphingomonas* (2.01%). For the larvae parasitizing *Q. acutissima*, the symbiotic microbiota consisted of 11 genera, with *Pseudomonas* (75.56%) as the dominant genus, followed by *Rickettsia* (4.12%). Additional genera identified included *Aquabacterium* (2.17%), *Sphingomonas* (2.01%), and *Acinetobacter* (1.85%).

**Figure 3:**
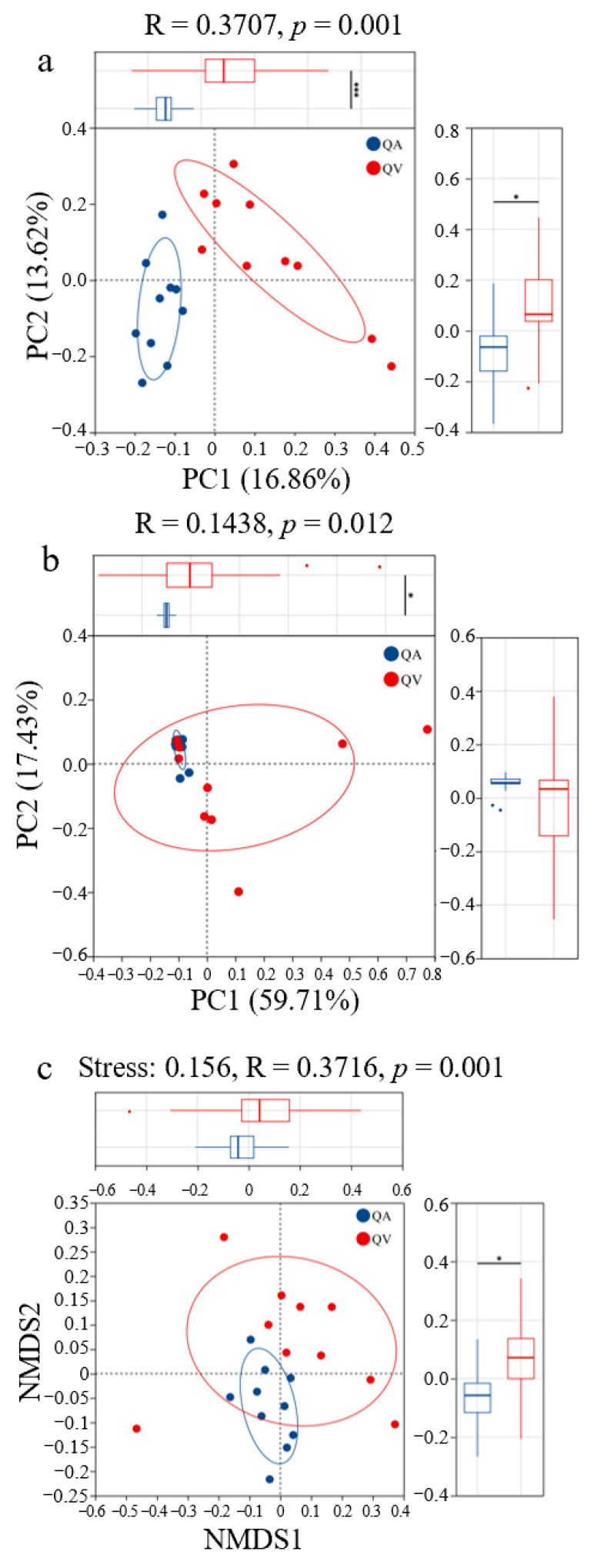
Beta-diversity of the symbiotic microbiota of gall wasp *Trichagalma acutissimae* parasitizing two oak species: (**a**): PCoA analysis based on the unweighted UniFrac distances; (**b**): PCoA analysis based on the weighted UniFrac distances, (**c**): Non-metric multidimensional scaling (NMDS). QA and QV stand for *Quercus acutissima* and *Q. variabilis*, respectively. * and *** stand for statistically significant at *p* < 0.05 and *p* < 0.001 level, respectively.

The PCoA analysis, based on the unweighted UniFrac distance, separated the symbiotic bacterial community composition between the larvae parasitizing the two hosts (ANOSIM = 0.3707, *p* = 0.001; Figure 3a). Moreover, there were significant differences in the symbiotic bacterial community composition between the larvae in the weighted UniFrac plot (ANOSIM = 0.1438; *p* = 0.012; Figure 3b). NMDS analysis revealed significant separation of samples along the NMDS2 axis (Stress = 0.156, R = 0.372, *p* = 0.001) (Figure 3c), further demonstrating a distinct clustering of the symbiotic bacteria communities in the *T. acutissimae* larvae parasitizing the two oak hosts.

### 3.3. Differences in Symbiotic Microbial Communities of T. acutissimae

**Figure 4:**
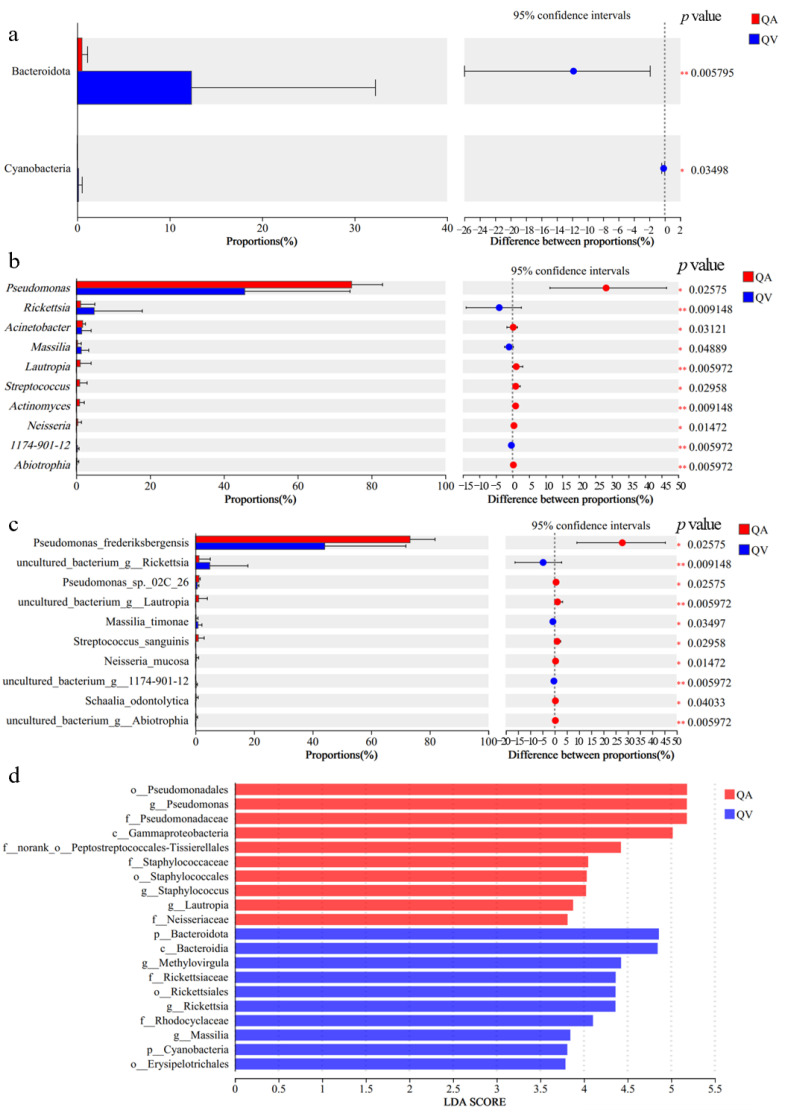
Comparison of symbiotic microbiota of gall wasp *Trichagalma acutissimae* parasitizing two oak species: (**a**): Wilcoxon rank-sum test bar plot at the phylum level; (**b**): Wilcoxon rank-sum test bar plot at the genus level; (**c**): Wilcoxon rank-sum test bar plot on species level; (**d**): LEfSe hierarchical tree. QA and QV stand for *Quercus acutissima* and *Q. variabilis*, respectively. * and ** stand for statistically significant at *p* < 0.05 and *p* < 0.01 level, respectively.

At the phylum level, the abundance of Bacteroidota and Cyanobacteria was significantly higher in *T. acutissimae* larvae parasitizing *Q. variabilis* than *Q. acutissma* (*p* = 0.006, *p* = 0.035) (Figure 4a). At the genus level, the abundance of *Pseudomonas* and *Acinetobacter* was markedly higher in *T. acutissimae* larvae parasitizing *Q. acutissma* than *Q. variabilis* (*p* = 0.026, *p* = 0.031). However, *Rickettsia* and *Massilia* were more abundant in *T. acutissimae* larvae parasitizing *Q. variabilis* (*p* = 0.009, *p* = 0.049) (Figure 4b). At the species level, *Pseudomonas frederiksbergensis*, the most abundant symbiotic bacteria, was much higher in *T. acutissimae* larvae parasitizing *Q. acutissma* than *Q. variabilis* (*p* = 0.026; Figure 4c).

The LEfSe analysis revealed that *Bacteroidota*, *Bacteroidia*, *Methlovirgula,* and *Rickettsiaceae* are relatively abundant in the *Q. variabilis* group, while *Pseudomonadales*, *Pseudomonas*, Pseudomonadaceae, and *Peptostreptococcales-Tissierellales* were more prevalent in the *Q. acutissma* group (Figure 4d).

**Figure 5:**
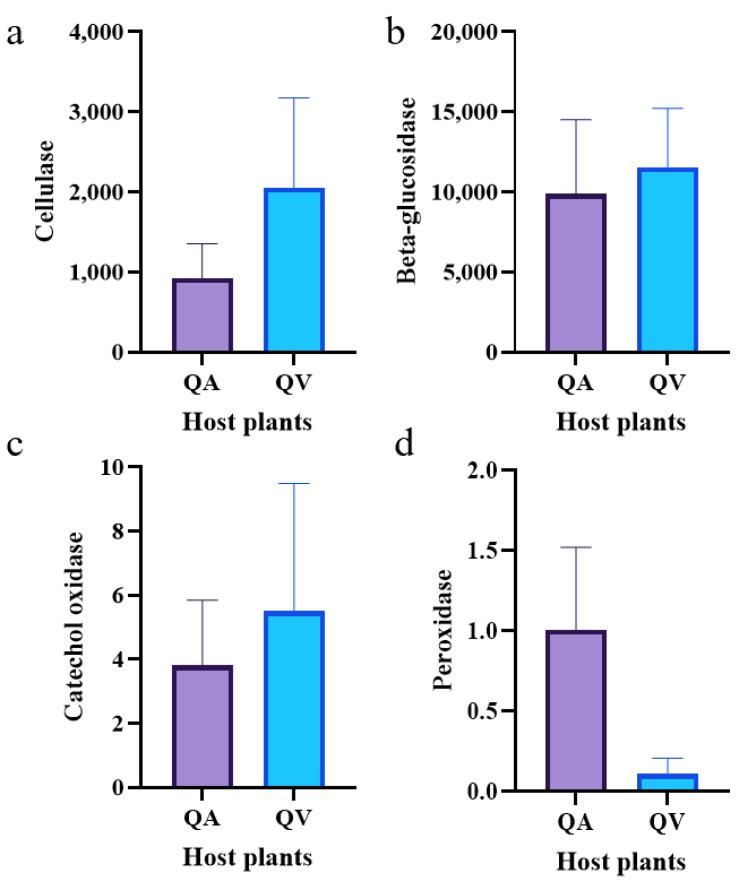
Abundance analysis of enzymes associated with tannin degradation of the gall wasp *Trichagalma acutissimae* parasitizing two oak species. (**a**): Cellulase; (**b**): Beta-glucosidase; (**c**): Catechol oxidase; (**d**): Peroxidase. QA and QV stand for *Quercus acutissima* and *Q. variabilis*, respectively.

Despite the differences in symbiotic bacteria in *T. acutissimae* larvae, we detected no significant difference in the abundance of enzymes responsible for tannin degradation, such as catechol oxidase, peroxidase, beta-glucosidase, and cellulase (Figure 5).

## 4. Discussion

As specialized symbionts, microorganisms play pivotal roles in host growth, development, ecological adaptation, and evolutionary dynamics [1,37]. Our study elucidates how host plant divergence between *Q. variabilis* and *Q. acutissima* shapes the symbiotic microbial community in *T. acutissimae* larvae, offering novel insights into insect–microbe–plant tripartite interactions.

### 4.1. Host-Driven Microbial Diversity Patterns of T. acutissimae

The insect symbiotic microbiome can be influenced by various factors, including diet and environmental conditions [38,39,40]. The diet of insects significantly shapes their gut microbial communities, with different diets promoting the proliferation of specific microbial populations, thereby enhancing digestion and nutrient utilization. Host dietary sources have been shown to significantly influence the diversity, structural organization, and compositional profiles of gut microbiota across various insect species [41]. For instance, larvae of *Ostrinia furnacalis* feeding on transgenic *Bt* maize exhibit a marked increase in the abundance of *Enterococcus* species within their gut microbiota. These bacterial communities contribute to insect resistance by degrading the *Bt* toxin protein Cry1Ab [42]. Similarly, variations in pollen sources substantially alter the abundance of core gut bacteria such as *Snodgrassella alvi* and *Gilliamella apicola* in *Apis mellifera*, thereby affecting their metabolic capacity for polysaccharides [43]. Previous studies have discovered that the symbiotic bacteria composition of *Trichoplusia ni* (Hubner) altered by diet may influence its polyphagous behavior [44]. But in the study of *Cnaphalocrocis medinalis*, the alpha diversity of gut microbiota was not affected by the host plant [15]. Our study investigates the composition of the symbiotic microbial community in larvae of *T. acutissimae* feeding on different host plants. Larvae parasitizing *Q. variabilis* exhibited significantly higher Shannon indices, but lower Simpson indices compared to *Q. acutissima*-fed counterparts, suggesting enhanced species evenness in the *Q. variabilis*-derived communities. Despite the temporal gap possibly affecting the observed microbiome differences, our findings suggest that *Pseudomonas* is a key factor contributing to the differences in symbiotic bacteria of *T. acutissimae* infesting two different oak species. The tannin contents in the fresh leaves of *Q. variabilis* and *Q. acutissima* are different, implying that tannin levels may influence the symbiotic bacteria of the wasp larvae [45]. This divergence likely reflects differential nutritional constraints imposed by host foliar chemistry, consistent with documented diet–microbiome interactions in holometabolous insects. To further verify that the wasps inducing galls on the two oak species belong to the same species, we systematically selected cynipid samples from both *Quercus variabilis* and *Q. acutissima* for molecular analysis. The COI gene was successfully sequenced, assembled, and compared with reference sequences retrieved from the NCBI database. The assembled COI sequences exhibited high similarity (>99%) to those of the same cynipid species deposited in NCBI, thereby confirming that all analyzed individuals indeed belong to the same species. Consequently, we conclude that the observed microbiome differences are unlikely attributable to genetic divergence among the cynipid individuals.

### 4.2. Phylum-Level Adaptations of T. acutissimae to Host Chemistry

Given that a single bacterial genus can harbor extensive phylogenetic and functional diversity [46], we explored the flora at the phylum level. Proteobacteria dominated larval microbiota in both host groups, aligning with patterns observed in *Ceratitis capitata* and other phytophagous insects [47,48,49,50]. In bacterial communities associated with other hymenopteran insects, such as the honey bee *Apis cerana* and *Leptocybe invasa*, Proteobacteria also dominate their gut bacterial community [51,52]. These results indicated that Proteobacteria are ubiquitous across a wide range of insect-associated bacterial communities. Previous studies have demonstrated that Proteobacteria are associated with carbohydrate digestion and play a key role in degrading flavonoids and tannins, which are important secondary metabolites plants [52,53], and there are notable differences in total soluble sugar content between the leaves of *Q. variabilis* and *Q. acutissima* [54]. Therefore, it is reasonable to infer that the disparity in Proteobacteria abundance between the larvae parasitizing *Q. variabilis* and *Q. acutissima* may be attributed to the differing carbohydrate contents in these two oak species.

### 4.3. Genus-Level Functional Specialization of T. acutissimae

At the genus level, *Pseudomonas* was identified as a dominant genus in larvae parasitizing both *Q. variabilis* and *Q. acutissima*, but its abundance was significantly higher in *Q. acutissima* compared to *Q. variabilis*. *Pseudomonas*, a member of Proteobacteria, is often considered pathogenic [55]. Some species of *Pseudomonas* have been identified in gall midges, gall aphid, gall sawflies, and psyllid [56,57,58]. Recently, *Pseudomonas* was identified from the bacterial communities as a predominant genus in chestnut tree galls induced by *Dryocosmus kuriphilus* [59]. In these studies, *Pseudomonas* was shown to be able to synthesize auxin and cytokinin to regulate the relationship between plants and gall worms. However, in our study, *Pseudomonas* may be attributed to high tannin content in the host plant. Research has shown that *Pseudomonas* can produce tannase to degrade tannins, thereby aiding hosts in digesting high-tannin diets and assisting insects in detoxifying or tolerating tannins in their food [49]. *Pseudomonas* predominated in the *Q. acutissima*-fed larvae, potentially facilitating tannin detoxification through tannase production. This functional advantage corresponds to the marginally lower tannin content of *Q. variabilis*, supporting the role of Proteobacteria in carbohydrate metabolism. This nutritional specialization may confer adaptive advantages for nutrient extraction from host-specific phloem sap.

*Apibacter*, known for its role in resisting parasite invasion in bees [60], showed QV-specific enrichment, possibly linked to earlier gall formation timelines that necessitate enhanced antiparasitic defenses. *Rickettsia* emerged as a conserved subdominant taxon, potentially influencing asexual reproduction and environmental stress resistance through mechanisms observed in other hymenopteran systems [61,62]. Additionally, *T. acutissimae* has an asexual generation stage [63], and Rickettsia, being a relatively abundant genus in its gut microbiota, may also impact reproduction and stress resistance. However, current research on this bacterium primarily focuses on plants, and its effects on *T. acutissimae* require further systematic investigation.

*Wolbachia* symbiotic bacteria are often found within the gall wasp [64], playing significant roles in various aspects of the gall wasp’s life cycle and survival strategies. Although the interaction between *Wolbachia* and the gall wasp is a complex and intriguing biological phenomenon [65,66,67], we showed no evidence of *Wolbachia* in bacterial symbionts of *T. acutissimae* in our study. *Wolbachia* is maternally transmitted via cytoplasmic inheritance through eggs and consequently cannot survive beyond the lifespan of a male host [68]. This may partially explain why *Wolbachia* is not detected in the asexual generation of *T. acutissimae*.

### 4.4. Result Supporting the Hypothesis of “Functional Redundancy”

Despite compositional differences, PICRUSt2 predictions revealed conserved metabolic profiles between the two larva groups. These metabolic profiles, which are crucial indicators of the physiological activities within the larvae, may provide valuable insights into their underlying biological processes. No significant difference was observed in the abundance of four tannin-degrading enzymes between the two sample groups. Tannins are complex polyphenolic compounds commonly found in plants, and their degradation is an important process for many insects that feed on tannin-rich plant materials. The four tannin-degrading enzymes under study play specific roles in breaking down tannins into more manageable components. The lack of significant difference in their abundance suggests that both larva groups may have similar strategies for dealing with tannins in their diet, regardless of other differences in their symbiotic microbiota or environmental factors.

Previous studies have demonstrated that many Lepidoptera insects harbor genes capable of encoding enzymes with related functionalities, which may contribute to the stability of enzyme abundances in gall-forming worms [69,70]. These genes not only allow the insects to adapt to different host plants but also play a role in maintaining the internal physiological balance. The stability of enzyme abundances is crucial for the normal functioning of the insects’ metabolic pathways, ensuring that they can efficiently process nutrients and respond to changes in their environment. Other studies have demonstrated that oak gall worms may have evolved conserved enzyme systems to adapt to the chemical composition of diverse host plants, thereby reflecting their adaptation strategy to multiple hosts. Oak gall worms encounter a wide range of chemical compounds in different oak species, and their conserved enzyme systems enable them to break down and utilize the available nutrients effectively. This adaptation strategy allows them to thrive on various oak hosts, increasing their ecological success and distribution.

In the present study, despite differences in the symbiotic microbiota composition between the larvae parasitizing *Q. variabilis* and *Q. acutissima*, no significant difference was observed in the abundance of enzymes. The differences in microbiota composition between the two larval groups may be due to various factors, such as differences in the microhabitats of the host plants, the feeding behaviors of the larvae, or the genetic characteristics of the larvae themselves. However, the fact that the enzyme abundances remained similar suggests that other factors, perhaps genetic regulation or compensatory mechanisms within the larvae, are at play in maintaining a consistent level of enzyme activity, regardless of the microbiota differences [71]. This finding further emphasizes the complexity of the interactions between insects, their symbiotic microbiota, and their host plants, and provides new avenues for future research to better understand these relationships.

## 5. Conclusions

Our study explored the intricate world of *T. acutissimae* inducing succulent galls on the leaves of two oak species, *Q.* *variabilis* and *Q. acutissima*. Our findings demonstrate a significant divergence in symbiotic microbiota composition between larvae infesting these two different oak species. The host identity of the gall-inducing wasp, along with its associated symbiotic microbiota, may play an important role in the adaptation and resistance mechanisms of various oak species. The complex interplay among these factors is expected to govern the survival and ecological dynamics of oak species within their respective environments. This investigation into the host plant species of the gall wasp and the characteristics of its symbiotic microbiota offers valuable insights into the underlying mechanisms driving the adaptation and resistance capabilities of different oak species.

## Data Availability

Raw data of 16S rRNA gene high throughput sequencing are available in the NCBI Sequence Read Archive (Bio-Project ID: PRJNA 1244591).

## Supplementary Materials

The following supporting information can be downloaded at: https://www.mdpi.com/article/10.3390/insects16070652/s1, Figure S1. Spherical galls induced by Trichagalma acutissimae on the oak leaves (a) and the longitudinal section of a fresh gall containing a larva in the inner capsule (b). Figure S2. Pan/Core/rarefraction-coverage analysis of the symbiotic microbiota of gall wasp Trichagalma acutissimae parasitizing two oak species: (a): Pan analysis; (b): Core analysis; (c): rarefraction-coverage analysis.

## References

[1] Wang X., Sun S., Yang X., Cheng J., Wei H., Li Z., Michaud J.P., Liu X. (2020). Variability of gut microbiota across the life cycle of *Grapholita molesta* (Lepidoptera: Tortricidae). Front. Microbiol..

[2] Zhang Y., Xu H., Tu C., Han R., Luo J., Xu L. (2024). Enhanced capacity of a leaf beetle to combat dual stress from entomopathogens and herbicides mediated by associated microbiota. Integr. Zool..

[3] Philipp E., Moran N.A. (2013). The gut microbiota of insects—diversity in structure and function. FEMS Microbiol. Rev..

[4] Bai S., Yao Z., Raza M.F., Cai Z., Zhang H. (2021). Regulatory mechanisms of microbial homeostasis in insect gut. Insect Sci..

[5] Sun H., Li H., Zhang X., Liu Y., Chen H., Zheng L., Zhai Y., Zheng H. (2023). The honeybee gut resistome and its role in antibiotic resistance dissemination. Integr. Zool..

[6] Prasad R.K., Chatterjee S., Sharma S., Mazumder P.B., Vairale M.G., Raju P.S. (2018). Insect gut bacteria and their potential application in degradation of lignocellulosic biomass: A review. Bioremediation: Applications for Environmental Protection and Management.

[7] Storelli G., Defaye A., Erkosar B., Hols P., Leulier F. (2011). *Lactobacillus plantarum* promotes drosophila systemic growth by modulating hormonal signals through TOR-Dependent nutrient sensing. Cell Metab..

[8] Erkosar B., Kolly S., van der Meer J.R., Kawecki T.J., Lemaitre B. (2017). Adaptation to chronic nutritional stress leads to reduced sependence on microbiota in drosophila melanogaster. Mbio.

[9] Bing X.L., Gerlach J., Loeb G., Buchon N. (2018). Nutrient-Dependent impact of microbes on *Drosophila suzukii* development. Mbio.

[10] Yun J.-H., Roh S.W., Whon T.W., Jung M.-J., Kim M.-S., Park D.-S., Yoon C., Nam Y.-D., Kim Y.-J., Choi J.-H. (2014). Insect gut bacterial diversity determined by environmental habitat, diet, developmental stage, and phylogeny of host. Appl. Environ. Microbiol..

[11] Iltis C., Tougeron K., Hance T., Louâpre P., Foray V. (2021). A perspective on insect–microbe holobionts facing thermal fluctuations in a climate-change context. Environ. Microbiol..

[12] Wang Y., Wang L., Li D., Chen Z., Luo Y., Zhou J., Luo B., Yan R., Liu H., Wang L. (2024). Advancements in the impact of insect gut microbiota on host feeding behaviors. Genes.

[13] Lou Y., Wang G., Zhang W., Xu L. (2025). Adaptation strategies of insects to their environment by collecting and utilizing external microorganisms. Integr. Zool..

[14] Lyte M. (2014). Microbial endocrinology and the microbiota-gut-brain axis. AEMB.

[15] Yang Y., Liu X., Xu H., Liu Y., Lu Z. (2022). Effects of host plant and insect generation on shaping of the gut microbiota in the rice leaffolder, *Cnaphalocrocis medinalis*. Front. Microbiol..

[16] Lü J., Guo W., Chen S., Guo M., Qiu B., Yang C., Lian T., Pan H. (2019). Host plants influence the composition of the gut bacteria in *Henosepilachna vigintioctopunctata*. PLoS ONE.

[17] Zhang S., Shu J., Xue H., Zhang W., Zhang Y., Liu Y., Fang L., Wang Y., Wang H., Heck M. (2020). The gut microbiota in *Camellia* weevils are influenced by plant secondary metabolites and contribute to saponin degradation. mSystems.

[18] Chen Y.-P., Li Y.-H., Sun Z.-X., Du E.-W., Lu Z.-H., Li H., Gui F.-R. (2022). Effects of host plants on bacterial community structure in larvae midgut of *Spodoptera frugiperda*. Insects.

[19] Jing T.-Z., Qi F.-H., Wang Z.-Y. (2020). Most dominant roles of insect gut bacteria: Digestion, detoxification, or essential nutrient provision?. Microbiome.

[20] Adair K.L., Douglas A.E. (2017). Making a microbiome: The many determinants of host-associated microbial community composition. Curr. Opin. Microbiol..

[21] Akami M., Njintang N.Y., Gbaye O.A., Andongma A.A., Rashid M.A., Niu C.-Y., Nukenine E.N. (2019). Gut bacteria of the cowpea beetle mediate its resistance to dichlorvos and susceptibility to *Lippia adoensis* essential oil. Sci. Rep..

[22] Frago E., Dicke M., Godfray H.C.J. (2012). Insect symbionts as hidden players in insect–plant interactions. Trends Ecol. Evol..

[23] Hansen A.K., Moran N.A. (2014). The impact of microbial symbionts on host plant utilization by herbivorous insects. Mol. Ecol..

[24] Akami M., Andongma A.A., Zhengzhong C., Nan J., Khaeso K., Jurkevitch E., Niu C.-Y., Yuval B. (2019). Intestinal bacteria modulate the foraging behavior of the oriental fruit fly *Bactrocera dorsalis* (Diptera: Tephritidae). PLoS ONE.

[25] Lv D., Liu X., Dong Y., Yan Z., Zhang X., Wang P., Yuan X., Li Y. (2021). Comparison of gut bacterial communities of fall armyworm (*Spodoptera frugiperda*) reared on different host plants. Int. J. Mol. Sci..

[26] Wang H., Jin L., Zhang H. (2011). Comparison of the diversity of the bacterial communities in the intestinal tract of adult *Bactrocera dorsalis* from three different populations. J. Appl. Microbiol..

[27] Jang S., Kikuchi Y. (2020). Impact of the insect gut microbiota on ecology, evolution, and industry. Curr. Opin. Insect Sci..

[28] Yang Z.-W., Luo J.-Y., Men Y., Liu Z.-H., Zheng Z.-K., Wang Y.-H., Xie Q. (2023). Different roles of host and habitat in determining the microbial communities of plant-feeding true bugs. Microbiome.

[29] Guo C., Peng X., Zheng X., Wang X., Wang R., Huang Z., Yang Z. (2020). Comparison of bacterial diversity and abundance between sexes of *Leptocybe invasa* Fisher & La Salle (Hymenoptera: Eulophidae) from China. PeerJ.

[30] Xue S., Zhang Y., Gao S., Lu S., Wang J., Zhang K. (2020). Mitochondrial genome of *Trichagalma acutissimae* (Hymenoptera: Cynipoidea: Cynipidae) and phylogenetic analysis. Mitochondrial DNA Part B.

[31] Michell C.T., Nyman T. (2021). Microbiomes of willow-galling sawflies: Effects of host plant, gall type, and phylogeny on community structure and function. Genome.

[32] Rinke C., Schwientek P., Sczyrba A., Ivanova N.N., Anderson I.J., Cheng J.-F., Darling A., Malfatti S., Swan B.K., Gies E.A. (2013). Insights into the phylogeny and coding potential of microbial dark matter. Nature.

[33] Shi W., Syrenne R., Sun J.Z., Yuan J.S. (2010). Molecular approaches to study the insect gut symbiotic microbiota at the ‘omics’ age. Insect Sci..

[34] Yang Z.-W., Men Y., Zhang J., Liu Z.-H., Luo J.-Y., Wang Y.-H., Li W.-J., Xie Q. (2021). Evaluation of sample preservation approaches for better insect microbiome research according to next-generation and third-generation sequencing. Microb. Ecol..

[35] Zhou J., Fong J.J. (2021). Strong agricultural management effects on soil microbial community in a non-experimental agroecosystem. Appl. Soil Ecol..

[36] Segata N., Izard J., Waldron L., Gevers D. (2011). Metagenomic biomarker discovery and explanation. Genome Biol..

[37] Liu Y., Shen Z., Yu J., Li Z., Liu X., Xu H. (2020). Comparison of gut bacterial communities and their associations with host diets in four fruit borers. Pest Manag. Sci..

[38] Jones A.G., Mason C.J., Felton G.W., Hoover K. (2019). Host plant and population source drive diversity of microbial gut communities in two polyphagous insects. Sci. Rep..

[39] Sugio A., Dubreuil G., Giron D., Simon J.-C. (2015). Plant–insect interactions under bacterial influence: Ecological implications and underlying mechanisms. J. Exp. Bot..

[40] Kikuchi Y., Hayatsu M., Hosokawa T., Nagayama A., Tago K., Fukatsu T. (2012). Symbiont-mediated insecticide resistance. Proc. Natl. Acad. Sci. USA.

[41] Li G., Sun J., Meng Y., Yang C., Chen Z., Wu Y., Tian L., Song F., Cai W., Zhang X. (2022). The impact of environmental habitats and diets on the gut microbiota diversity of true bugs (Hemiptera: Heteroptera). Biology.

[42] Xu T., Wang Y., Wang Y., Bi S., Hu B., Hu F., Xu L. (2023). Comparison of gut microbial community between Bt-Resistant and susceptible strains of *Ostrinia furnacalis*. Agronomy.

[43] Bueren E.K., Weinheimer A.R., Aylward F.O., Hsu B.B., Haak D.C., Belden L.K. (2023). Characterization of prophages in bacterial genomes from the honey bee (*Apis mellifera*) gut microbiome. PeerJ.

[44] Leite-Mondin M., Dilegge M.J., Manter D.K., Weir T.L., Vivanco J.M. (2021). The gut microbiota composition of *Trichoplusia ni* is altered by diet and may infuence its polyphagous behavior. Sci. Rep..

[45] Shozo T. (2005). Discrimination of hybrids between *Quercus variabilis* and *Q. acutissima* by using stellate hairs, and analysis of the hybridization zone in the Chubu District of central Japan. J. Phytogeogr. Taxon..

[46] Coenye T., Vandamme P. (2003). Diversity and significance of Burkholderia species occupying diverse ecological niches. Environ. Microbiol..

[47] Behar A., Yuval B., Jurkevitch E. (2008). Gut bacterial communities in the Mediterranean fruit fly (*Ceratitis capitata*) and their impact on host longevity. J. Insect Physiol..

[48] Schloss P.D., Delalibera I., Handelsman J., Raffa K.F. (2006). Bacteria associated with the guts of two wood-boring beetles: *Anoplophora glabripennis* and *Saperda vestita* (Cerambycidae). Environ. Entomol..

[49] Guo S.-h., Yi X.-f. (2019). Gut bacterial composition of two *Curculio* species and their adaptation to high-tannin food. Acta Microbiol. Sin..

[50] Kou R.-M., Li Y., Dou F.-Y., Zhou Z.-Y., Huang D.-Y. (2021). Diversity and differences of gut bacterial communities in different instar larvae and diapause prepupae of *Colletes gigas* (Hymenoptera: Colletidae). Acta Entomol. Sin..

[51] Ahn J.-H., Hong I.-P., Bok J.-I., Kim B.-Y., Song J., Weon H.-Y. (2012). Pyrosequencing analysis of the bacterial communities in the guts of honey bees *Apis cerana* and *Apis mellifera* in Korea. J. Microbiol..

[52] Liu Y., Xu L., Zhang Z., Huang Z., Fang D., Zheng X., Yang Z., Lu M. (2021). Isolation, identification, and analysis of potential functions of culturable bacteria associated with an invasive gall wasp, *Leptocybe invasa*. Microb. Ecol..

[53] Flint H.J., Scott K.P., Duncan S.H., Louis P., Forano E. (2012). Microbial degradation of complex carbohydrates in the gut. Gut Microbes.

[54] Yin S., Sun X. (2002). Studies on the interrelationship among *Oligonychus ununguis*, host plants and *Amblyseius finlandicus*. III. Relationship between chemical component of host plants and development of *Oligonychus ununguis*. Scient. Silvae Sin..

[55] Jander G., Rahme L.G., Ausubel F.M. (2000). Positive correlation between virulence of *Pseudomonas aeruginosa* mutants in mice and insects. J. Bacteriol..

[56] Tanaka Y., Okada K., Asami T., Suzuki Y. (2013). Phytohormones in Japanese mugwort gall induction by a gall-inducing gall midge. Biosci. Biotechnol. Biochem..

[57] Takei M., Yoshida S., Kawai T., Hasegawa M., Suzuki Y. (2015). Adaptive significance of gall formation for a gall-inducing aphids on Japanese elm trees. J. Insect Physiol..

[58] Kai S., Kumashiro S., Adachi S., Suzuki Y., Shiomi Y., Matsunaga K., Gyoutoku N., Asami T., Tokuda M. (2017). Life history of *Stenopsylla nigricornis* (Hemiptera: Psylloidea: Triozidae) and phytohormones involved in its gall induction. Arthropod-Plant Interact..

[59] Yang X., Hui Y., Zhu D., Zeng Y., Zhao L., Yang X., Wang Y. (2022). The diversity of bacteria associated with the invasive gall wasp *Dryocosmus kuriphilus*, its galls and a specialist parasitoid on chestnuts. Insects.

[60] Kwong W.K., Moran N.A. (2016). *Apibacter* adventoris gen. nov., sp. nov., a member of the phylum *Bacteroidetes* isolated from honey bees. Int. J. Syst. Evol. Microbiol..

[61] Kageyama D., Narita S., Watanabe M. (2012). Insect sex determination manipulated by their endosymbionts: Incidences, mechanisms and implications. Insects.

[62] Himler A.G., Adachi-Hagimori T., Bergen J.E., Kozuch A., Kelly S.E., Tabashnik B.E., Chiel E., Duckworth V.E., Dennehy T.J., Zchori-Fein E. (2011). Rapid spread of a bacterial symbiont in an invasive whitefly is driven by fitness benefits and female bias. Science.

[63] Wang J., Wang X., Zhang K., Wu S. (2017). Factors influencing the temporal and spatial population dynamics of *Trichagalma cutissimae* (Hymenoptera: Cynipidae). J. Appl. Entomol..

[64] Boivin T., Henri H., Vavre F., Gidoin C., Veber P., Candau J.N., Magnoux E., Roques A., Auger-Rozenberg M.A. (2014). Epidemiology of asexuality induced by the endosymbiotic *Wolbachia* across phytophagous wasp species: Host plant specialization matters. Mol. Ecol..

[65] Zhao G.-Z., Zhu T.-R., Zeng Y., Zhu D.-H. (2021). *Wolbachia* infection in six species of gall wasps and their parasitoids. J. Asia Pac. Entomol..

[66] Taprogge M., Grath S. (2024). Modelling suggests *Wolbachia*-induced cytoplasmic incompatibility in oak gall wasps with cyclical parthenogenesis. J. Evol. Biol..

[67] Watts J. (2023). *Wolbachia* Infection in Gall Associated Insect Communities in Illinois and Indiana. Master’s Thesis.

[68] Pijls J.W., van Steenbergen H.J., van Alphen J.J. (1996). Asexuality cured: The relations and differences between sexual and asexual *Apoanagyrus diversicornis*. Heredity.

[69] Wybouw N., Dermauw W., Tirry L., Stevens C., Grbić M., Feyereisen R., Van Leeuwen T. (2014). A gene horizontally transferred from bacteria protects arthropods from host plant cyanide poisoning. eLife.

[70] Sun B., Xiao J., He S., Liu L., Murphy R., Huang D. (2013). Multiple ancient horizontal gene transfers and duplications in *Lepidopteran species*. Insect Mol. Biol..

[71] Louca S., Polz M.F., Mazel F., Albright M.B., Huber J.A., O’Connor M.I., Ackermann M., Hahn A.S., Srivastava D.S., Crowe S.A. (2018). Function and functional redundancy in microbial systems. Nat. Ecol. Evol..

