# Supplementary material for: Host Plant Dependence of the Symbiotic Microbiome of the Gall-Inducing Wasp Trichagalma acutissimae
Source: Insects. 2025 Jun 23;16(7):652. doi: 10.3390/insects16070652 (PMC12294959; doi:10.3390/insects16070652)
Supplement: Supplementary file 1 [file insects-16-00652-s001.zip › insects-3628795-supplementary.pdf]

insects

MDPI

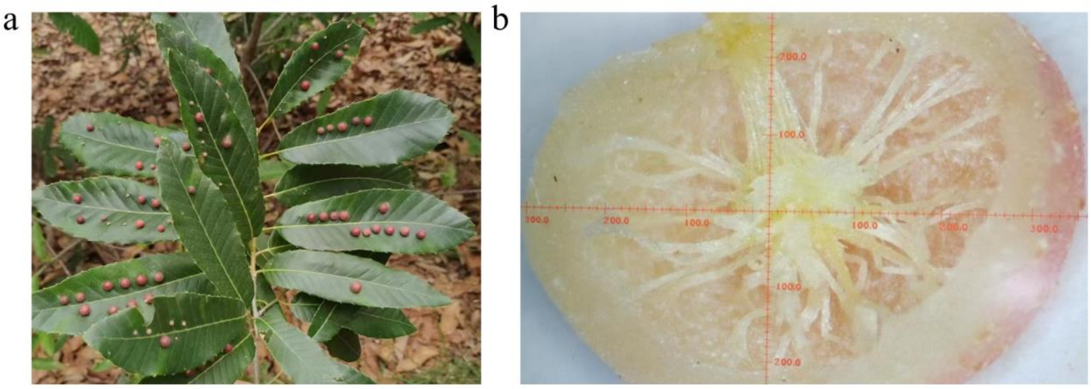

Figure S1. Spherical galls induced by *Trichagalma acutissimae* on the oak leaves (a) and the longitudinal section of a fresh gall containing a larva in the inner capsule (b).

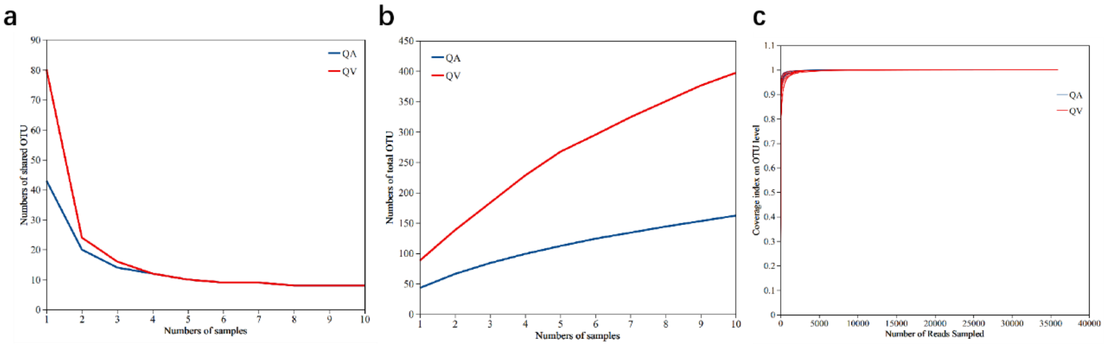

Figure S2. Pan / Core / rarfraction-coverage analysis of the symbiotic microbiota of gall wasp *Trichagalma acutissimae* parasitizing two oak species: (a): Pan analysis; (b): Core analysis; (c): rarfraction-coverage analysis.
